# Factors Influencing NO_2_ Adsorption/Reduction on Microporous Activated Carbon: Porosity vs. Surface Chemistry

**DOI:** 10.3390/ma11040622

**Published:** 2018-04-18

**Authors:** Imen Ghouma, Mejdi Jeguirim, Lionel Limousy, Najoua Bader, Abdelmottaleb Ouederni, Simona Bennici

**Affiliations:** 1 Institut de Science des Matériaux de Mulhouse, IS2M-UMR7361-CNRS-UHA, 15 Rue Jean Starcky, 68057 Mulhouse, France; imenghouma83@gmail.com (I.G.); lionel.limousy@uha.fr (L.L.); simona.bennici@uha.fr (S.B.); 2 Laboratoire Génie des Procédés et Systèmes Industriels, Université de Gabès, St Omar El Khattab, 6029 Gabes, Tunisia; najoua.bader@gmail.com (N.B.); mottaleb.ouederni@enig.rnu.tn (A.O.)

**Keywords:** activated carbon, olive stones, textural properties, surface chemistry, NO_2_ adsorption, chemisorption, physisorption

## Abstract

The textural properties and surface chemistry of different activated carbons, prepared by the chemical activation of olive stones, have been investigated in order to gain insight on the NO_2_ adsorption mechanism. The parent chemical activated carbon was prepared by the impregnation of olive stones in phosphoric acid followed by thermal carbonization. Then, the textural properties and surface chemistry were modified by chemical treatments including nitric acid, sodium hydroxide and/or a thermal treatment at 900 °C. The main properties of the parent and modified activated carbons were analyzed by N_2_-adsorption, scanning electron microscopy (SEM), and Fourier transform infrared spectroscopy (FTIR) techniques, in order to enlighten the modifications issued from the chemical and thermal treatments. The NO_2_ adsorption capacities of the different activated carbons were measured in fixed bed experiments under 500 ppmv NO_2_ concentrations at room temperature. Temperature programmed desorption (TPD) was applied after adsorption tests in order to quantify the amount of the physisorbed and chemisorbed NO_2_. The obtained results showed that the development of microporosity, the presence of oxygen-free sites, and the presence of basic surface groups are key factors for the efficient adsorption of NO_2_.

## 1. Introduction

Nitrogen oxides (NO_x_) are among the most pollutant gases and largely contribute to acid rain formation and the depletion of the ozone layer. NO_x_ are also applied in various secondary processes, generating harmful molecules as ozone and acid compounds. Several processes were used for NO_x_ emissions treatment, including NO_x_ storage and reduction (NSR) and selective catalytic reduction (SCR) [1,2,3,4]. These methods are particularly suitable for the treatment of automotive exhaust gases, but they are expensive and result in technically complicated applications in industrial plants. Therefore, nowadays researches on NO_x_ control technologies are mainly focused on the identification of cheaper and more efficient elimination techniques.

Recently, the removal of NO_2_ at low temperatures using activated carbons has proven to be a promising technique. Several precursors such as sawdust pellets [5], bituminous coal [6], palm shell [7], coffee industry waste materials [8], date stones [9,10], and sewage sludge [11] have been used for AC production. The performance of these activated carbons were evaluated for the removal of NO_2_ at ambient temperature. The adsorption capacities ranged from 17 to 140 mg/g [5,6,7,8,9,10,11,12,13] depending on the activation procedure and the operating conditions for adsorption [14]. 

NO_2_ can be adsorbed on the activated carbon surface through physisorption, chemisorption, and/or reduction processes. In particular, previous investigations showed the formation of various surface complexes such as C-NO_2_, C-ONO, C-ONO_2_, and C-O [15,16,17]. These surface complexes are mainly formed on the external surface and they can be considered as new functional groups or as temporary activated species formed by the interaction of NO_2_ with the already existing surface oxygen groups [18].

Several studies investigated the role of oxygen surface groups on NO_2_ adsorption by activated carbon. Various treatments were applied in order to elaborate activated carbons with different porous textures as well as different amounts and the nature of surface oxygen groups. Belala et al. [9] studied the adsorption of NO_2_ at low temperature on an activated carbon prepared from date pits. In this study, the maximum adsorption capacity was about 107 mg/g. The authors observed that the development of porosity with an increasing time of activation favors the adsorption capacity of NO_2_. Belhachemi et al. [10] have compared the removal of NO_2_ using date pits activated carbon and modified commercialized activated carbon. Activated carbon prepared from date pits resulted in an efficient adsorbent, being characterized by adsorption capacities similar to those of commercial adsorbents. The maximum adsorption capacity reached was about 136 mg/g using the commercial activated carbon GAC. The authors reported that the adsorption capacities decrease by increasing the amount of acidic surface groups. Nowicki et al. [19] studied the effect of the treatment with urea to modify the textural properties and the acid–base character of the activated carbon surfaces. Moreover, they showed that the choice of the activation and modification procedure of coniferous tree sawdust produces activated carbons with high nitrogen dioxide adsorption capacity (reaching 69 mg NO_2_/g in wet conditions). The adsorption capacity seems to depend both on the textural properties and the acid–base character of the adsorbent surfaces. These observations were confirmed by Bazan et al. [13] that showed how activated carbons with low-developed surface area (varying from 2 to 206 m^2^/g with a clear basic surface character) can be effective for the removal of NO_2_ from the atmosphere.

In addition to the chemical modification of activated carbons, metals impregnation was studied in order to improve the adsorption capacity. Sager et al. [20] reported on the influence of modified activated carbons with CuO/ZnO on the adsorption of NO_2_ at room temperature. The authors have shown that the modification of activated carbon with 5 wt % CuO/ZnO leads to an increase of the NO_2_ adsorption capacity. The results have also shown that the efficiency of the sorbent can be more than doubled by increasing the metal oxide loading up to 20 wt %. In similar way, Yoo et al. [21] have studied an activated carbon filter soaked in water containing metals such as Cu and Mn. The results showed that the activated carbon loaded with various metals can efficiently reduce the NO_2_ and HONO concentrations in indoor air. The effect of the porous texture and the surface chemistry on the removal of NO_2_ at low concentration was also investigated by comparing the adsorption performances of three activated carbons prepared from olive stones using different activation routes [22].

During the interaction of NO_2_ with carbon materials, a significant amount of NO is emitted [23]. Therefore, it is important to find a way to limit NO_2_ reduction to NO during the adsorption step, when using activated carbon adsorbents. Xue et al. [24] studied the role of the surface properties of an activated carbon for the adsorption of NO. The results showed that a wet oxidation treatment can increase the amount of surface oxygen groups on the carbon surface, resulting in the enhancement of NO adsorption. These observations were confirmed by Bashkova et al. [25] in the removal of NO_2_ by wood-based activated carbons modified with urea and thermally treated at 950 °C. The authors found that the induced surface modifications have a positive effect on NO_2_ adsorption as well as on the adsorption of NO (product of NO_2_ reduction during the adsorption step).

Although the mechanism of NO_2_ adsorption over activated carbon has been largely reported in the literature, the role of the surface oxygen groups is still not clear. For this reason, the present work aims to elucidate the roles of textural properties and surface chemistry in the process of NO_2_ adsorption on activated carbons. For this purpose, a series of activated carbons have been prepared starting from the same precursor in order to have similar textural properties and different amounts and types of surface groups. Such an approach permits the correlation of the adsorption performances and mechanisms (physisorption, chemisorption) to the different properties of the activated carbons.

## 2. Materials and Methods

### 2.1. Synthesis of Chemically Activated Carbon

A parent chemically activated carbon was prepared from olive stones according to the optimized protocol previously reported [26]. Firstly, the raw precursor was washed with plenty of hot distilled water in order to obtain grains of olive stones with diameters between 1 and 3 mm. Then, a portion of the olive stones sample was soaked in an aqueous solution containing orthophosphoric acid (H_3_PO_4_ 50%, *w*/*w*) at the weight ratio (1:3). The suspension was stirred at 110 °C for 9 h. The filtered material was dried and carbonized under nitrogen flow at 170 °C for 30 min, and finally treated at 410 °C for 2 h 30 min. The resulting carbon, denoted as A, was then carefully washed with distilled water until the complete elimination of the acid. The preparation procedure ended by overnight drying at 110 °C.

### 2.2. Modification of the Surface Chemistry 

In order to prepare AC with different surface chemistry, various protocols were applied. The different protocols are described in the following sections.

#### 2.2.1. Wet Oxidation

A mass of 30 g of chemically activated carbon (A) was mixed with 250 mL of 1 M nitric acid aqueous solution and under reflux for 8 h. The resulting materials were filtered and carefully washed with distilled water until the filtered water pH value was approximately 7. The sample was labeled A-HNO_3_.

#### 2.2.2. NaOH Neutralization

A mass of 50 g of chemically activated carbon (A) was added to a 1 M solution of NaOH and refluxed for 3 h. Then, the activated carbon was filtered and largely washed with distilled water. Finally, it was dried overnight at 105 °C and labeled A-NaOH.

#### 2.2.3. Thermal Treatment

Sample A was left at 900 °C for 1 h under nitrogen flow of 10 NL h^−1^ and then cooled down to room temperature. This treatment was efficient for the removal of the surface oxygenated functional groups. The resultant sample was labelled A-TT. 

### 2.3. Morphological and Textural Properties Characterization

Scanning electron microscopy (Philips model FEI model Quanta 400 SEM, Amsterdam, The Netherlands) was used to analyze the morphology and the microscopic shape of the different activated carbons. A characterization of the pore structure of the activated carbon samples was made by measurement of the N_2_ adsorption isotherms using an automatic gas sorption analyzer (ASAP 2010, Micrometrics, Norcross, GA, USA). Prior to measurement the sample was outgassed at 120 °C under vacuum for 12 h to ensure a dry clean surface free from any loosely held adsorbed species. The specific surface area was calculated from the N_2_-adsorption isotherms applying the Brunauer–Emmett–Teller (BET) equation and this provided important information about structural features. The t-plot method was applied to calculate the micropores surface area and the micropores volume. 

### 2.4. Surface Chemistry Characterization

Different techniques were also applied for the analysis of the surface oxygen groups formed during the activation and the modification of the activated carbons.

#### 2.4.1. Temperature Programmed Desorption-Mass Spectroscopy (TPD-MS)

The surface chemistry of the samples was analyzed by temperature programmed desorption coupled with mass spectrometry (TPD-MS). The sample weighing 10 mg was placed in a quartz tube and heat-treated with a linear heating rate of 5 °C/min under vacuum. The material surface chemistry was evaluated in the temperature range 25–900 °C. The gases evolved during the heating process were continuously and quantitatively analyzed by a mass spectrometer. The desorption rate of each gas as a function of temperature was determined from the TPD analysis. The total amount of each gas released was computed by a time integration of the TPD curves.

#### 2.4.2. Fourier Transform Infrared Spectroscopy (FTIR)

Fourier transformed infrared spectroscopy (FTIR) was used to characterize the main functional surface groups of the activated carbon using a spectrometer FTIR (Jasco FT-IR 4100 series spectrophotometer with a diffuse reflectance accessory manufactured by PIKE Technologies, Madison WI, USA). Self-supported pellets of the various samples were obtained by mixing and pressing the activated carbon powders with finely divided spectroscopic grade KBr. A minimum of 30 scans was acquired for each spectra with a spectral resolution of 16 cm^−1^. During these analyses, similar amounts and thicknesses for all of the samples were considered in order to be able to compare the peak intensity of the different surface groups.

#### 2.4.3. Boehm Titration

The Boehm method is described as follows: 1 g of each sample was placed during 72 h, in 50 mL of 0.1 N solutions of: hydrochloric acid, sodium hydroxide, sodium carbonate, and sodium hydrocarbonate. Then, residual acid or base in each solution was titrated with HCl or NaOH. The number of acidic groups was determined on the assumption that NaOH neutralizes carboxyls, phenols and lactone groups, Na_2_CO_3_ neutralizes carboxyls and lactone groups, and NaHCO_3_ neutralizes only carboxyls. The basic group content was obtained from the amount of HCl that reacted with the carbon.

### 2.5. NO_2_ Adsorption Tests

**Figure 1:**
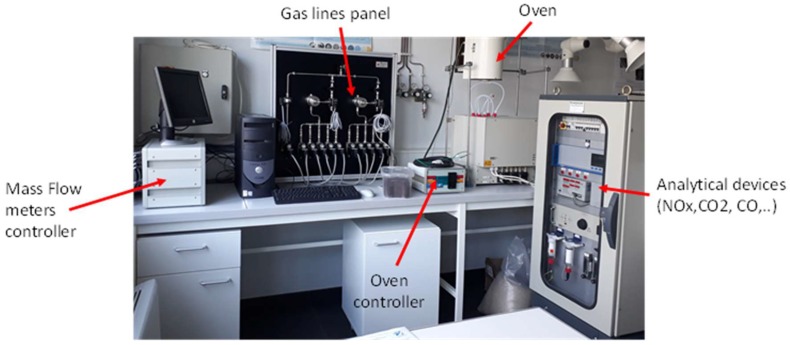
Photo of the Experimental bench used for the NO_2_ adsorption tests.

The NO_2_ adsorption experiments were performed in a fixed bed reactor presented in Figure 1. For each experiment, 100 mg of activated carbon was deposited on a fused silica frit placed in a vertical quartz reactor (with an internal diameter of 6 mm). The bed temperature was measured by a thermocouple placed at 1 mm above the surface of the AC sample. A gas stream mixture containing 500 ppmv of NO_2_ diluted in nitrogen stream was injected through the column of fixed bed of the adsorbent (AC). A constant gas flow rate of 20 NL h^−1^ was maintained by using BROOKS 5850 mass flow controllers (Seattle, WA, USA). Outlet NO, NO_2_, CO, and CO_2_ concentrations were continuously monitored by a ROSEMOUNT NGA 2000 detector (St. Louis, MO, USA), with a time of acquisition of 2 s. 

The amount of NO_2_ adsorbed was calculated according to Equations (1) and (2):

(1) $${\mathrm{NO}}_{2\mathrm{ads}}\left(t\right)\left(\frac{\mu \mathrm{mol}}{s}\right)=\left({\left[{\mathrm{NO}}_{2}\right]}_{\mathrm{inlet}}-\left({\left[{\mathrm{NO}}_{2}\right]}_{\mathrm{outlet}}+{\left[\mathrm{NO}\right]}_{\mathrm{outlet}}\right)\right)\times 10^{-6}\times Q/V_{M}$$

(2) $${\mathrm{NO}}_{2\mathrm{ads}}\left(\mathrm{mg}/g\right)=\int_{0}^{t}({\left[{\mathrm{NO}}_{2}\right]}_{\mathrm{ads}}\left(t\right)\times 10^{6}\times M_{{\mathrm{NO}}_{2}}/m_{\mathrm{CA}})\;\mathrm{dt}$$

where ${\mathrm{NO}}_{2\mathrm{ads}}\left(t\right)$ is the adsorbed rate of NO_2_ in µmol/s or the adsorption capacity of the AC (mg/g). NO_2inlet_ is the inlet NO_2_ concentration (ppmv). NO_2outlet_ and NO_outlet_ are, respectively, the outlet NO_2_ and NO concentrations (ppmv). Q corresponds to the gas flow rate (NL/s). V_M_ is the molar volume at normal conditions (22.4 L/mol). M_NO2_ is the molar mass of NO_2_ (46,000 mg/mol) and m_AC_ is the mass of activated carbon used for the adsorption test (g).

After exposure of the sample to the NO_2_ flow, the reactor was cooled down to room temperature and TPD analysis performed up to 900 °C at a heating rate of 5 °C/min under nitrogen atmosphere. The desorption curves showed peaks that can be assigned to the decomposition of the components adsorbed at the surface of the activated carbon [27]. The amounts of NO_2_ and NO desorbed during the TPD experiments were calculated according to Equations (3) and (4):

(3) $${\mathrm{NO}}_{2\mathrm{des}}\left(\text{µ}\mathrm{mol}/g\right)=\frac{D}{m_{\mathrm{CA}}\times V_{M}}\int_{0}^{t}({\left[{\mathrm{NO}}_{2}\right]}_{\mathrm{outlet}})\;\mathrm{dt}$$

(4) $${\mathrm{NO}}_{\mathrm{des}}\left(\text{µ}\mathrm{mol}/g\right)=\frac{D}{m_{\mathrm{CA}}\times V_{M}}\int_{0}^{t}({\left[\mathrm{NO}\right]}_{\mathrm{outlet}})\;\mathrm{dt}$$

From these calculations, the amount of NO released can be assumed to correspond to chemisorbed NO_2_, while the amount of NO_2_ released during the TPD can be related to the physisorbed fraction of NO_2_.

## 3. Results and Discussions

### 3.1. Activated Carbons Characterization 

#### 3.1.1. Morphological and Textural Properties

**Figure 2:**
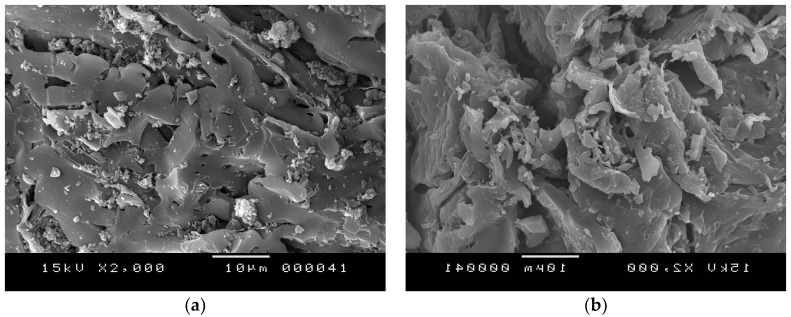
Scanning Electron Microscope (SEM) micrographs of sample two activated carbon (AC) samples with a magnification of × 2000: (**a**) A; (**b**) A-HNO_3_.

SEM images of samples A and A-HNO_3_ are presented in Figure 2a,b respectively. The samples display different surface morphologies, independent to the applied AC activation procedure, and performed with different acids and operating conditions.

Sample A, which was activated with H_3_PO_4_, presents an arrangement in tightly compacted sheets. The addition (or insertion) of phosphate groups drives a dilation process that, after the removal of the acid, leaves the matrix in an expanded state with an accessible pore structure [28]. The analysis of the modified activated carbon with HNO_3_ (Figure 2b) shows the conservation of the parent activated carbon morphology without a widening of the macro-porosity, even after 8 h of treatment. This is contrary to what was previously observed during oxidation treatments with increasing acid concentration, which usually led to the collapse of the carbon framework [29].

**Figure 3:**
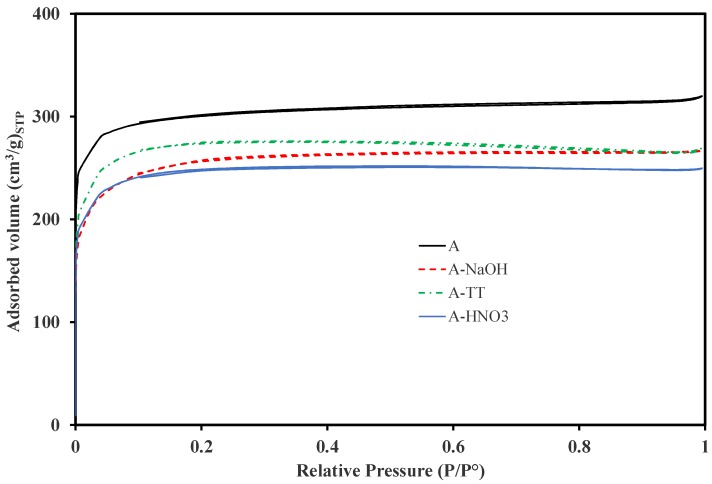
Nitrogen adsorption isotherms obtained at −196 °C for the different AC samples, before (A) and after the surface treatment (A-HNO_3_, A-NaOH and A-TT).

Figure 3 shows the nitrogen adsorption isotherms acquired at −196 °C for the different AC samples. All the ACs show a type-I isotherm according to the IUPAC classification, and typical for microporous materials. In addition, sample A shows the highest nitrogen uptake, corresponding to the highest surface area and the best developed porosity. All surface treatments performed on this sample led to a decreasing of the nitrogen uptake.

**Figure 4:**
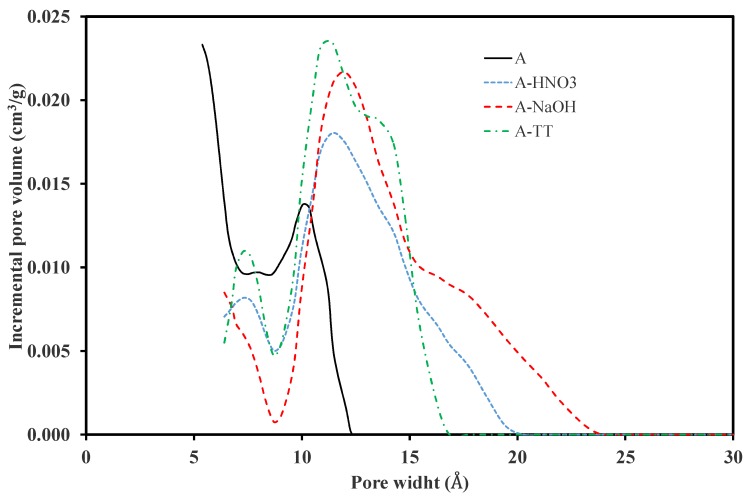
Pore size distribution of the different ACs estimated by nonlocal density functional theory (NLDFT) method.

The pore size distributions (PSDs) of the different samples were estimated using the nonlocal density functional theory (NLDFT) method. The results are presented in Figure 4. Sample A presents a mono-dimensional PSD centered in the ultramicropore range (maximum of the pore width distribution lower than 1 nm). All other samples, and in particular the NaOH–treated sample, display a wider PSD in the micropores range (pore width between 0.6 nm and 2.5 nm). This wide value range can be attributed to the enlargement of the micropores due to the destruction of the basal planes.

**Figure 5:**
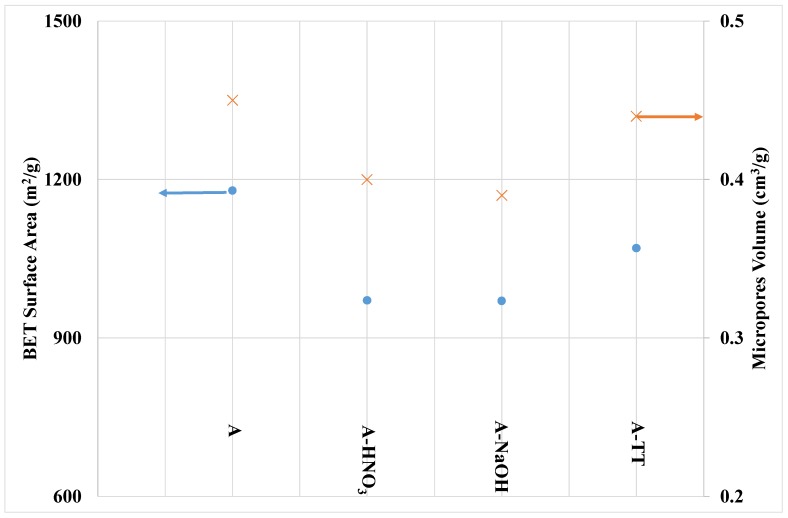
Brunauer–Emmett–Teller (BET) surface area and micropores volume (calculated by Horvath-Kawazoe method) for the different samples, deduced from N_2_ adsorption isotherms carried-out at −196 °C.

The textural properties of the carbon materials deduced from the nitrogen adsorption isotherms are reported in Figure 5. As expected, sample A has the highest surface area and the best developed internal porosity. The activated carbons are essentially microporous. In addition, all the surface treatments led to the decreasing of their pore volume and surface area. The surface treatments that most strongly impact the parent activated carbon porosity are those performed with nitric acid and NaOH. The decreasing of the microporous volume for the treated samples can be related or to newly created oxide functional groups that might block the access to the micropores, or to coalescence processes that involve parts of the micropores and are derived from the destruction of the micropore walls due to oxidation. Shim et al. [30] observed that, because of the high stability of the graphite basal planes, oxygen surface groups are expected to be located at the edges of the basal planes and are relatively weakly bonded to the carbon structure.

In contrast, the slight decrease in porosity observed after the thermal treatment is unexpected since the thermal treatment leads to the removal of more stable oxygen functional groups and porosity liberation. Various research groups have already reported on the increase of carbon porosity after a heat treatment [31,32,33,34]. In this study, the thermal treatment performed at 900 °C may be too high and therefore damage slightly the internal carbon porosity. 

#### 3.1.2. Surface Chemistry

**Figure 6:**
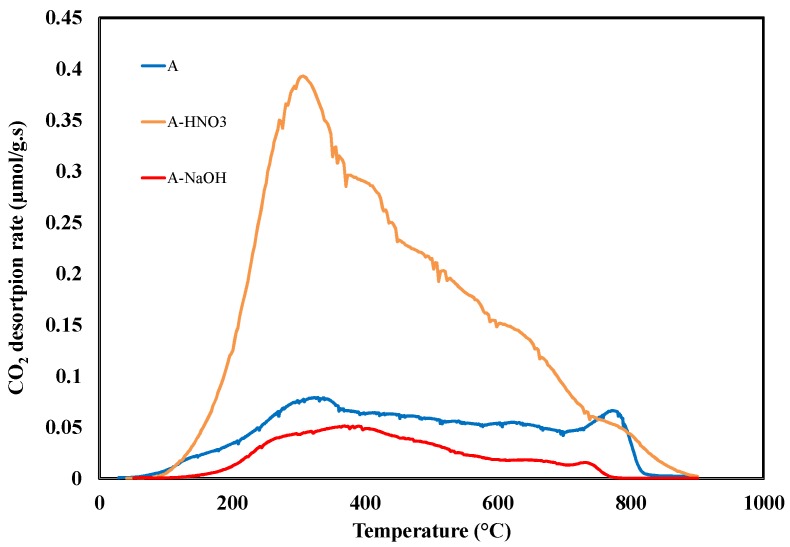
CO_2_ desorption profiles of sample A and samples of A treated by HNO_3_ or NaOH.

TPD–MS experiments provide the evolution of CO_2_ and CO emissions as a result of the decomposition of the oxygen functional groups present on the activated carbons surface. The determination of the amount of CO and CO_2_ emitted during TPD gives an estimation of the amount of surface oxygen groups present on the activated carbons. Moreover, different desorption temperatures correspond to the presence of different oxygen surface groups. The CO_2_ released at low temperatures (from 200 to 500 °C) derives from the decomposition of acid surface groups, while CO emissions are related to the decomposition of weak acidic, neutral and basic groups, which are more thermally stable and therefore emitted at higher temperatures (from 400 to 800 °C) [31,32]. The TPD profiles of CO_2_ from the various samples are shown in Figure 6. Clearly, the TPD-MS experiment carried out on sample A is characterized by the presence of CO_2_ emissions, which are attributed to the presence of acidic groups such as carboxylic, lactone, and anhydride. These oxygen surface groups were generated during the activation by phosphoric acid. The oxidation of carbon A by HNO_3_ increases significantly the amount of acidic groups as shown in Figure 6. In contrast, the CO_2_ emission decreases significantly by NaOH treatment but do not disappear completely. Such behavior is attributed to the neutralization of strong acidic groups such as carboxylic by NaOH. 

**Figure 7:**
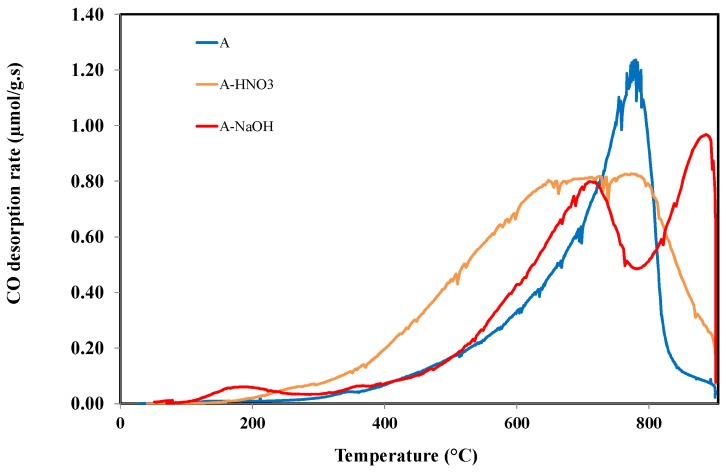
CO desorption profiles of sample A and samples of A treated by HNO_3_ or NaOH.

Figure 7 shows the TPD profiles of CO from the various samples studied. These profiles have different shapes in term of peaks and intensities. Therefore, it seems clear that the nature of the surface groups is different between the different samples. In particular, sample A shows a major peak between 400 and 800 °C, which is attributed to the anhydride, phenol, and ether groups. The sample A-HNO_3_ displays one large peak with an increase in the amount of CO below 600 °C. This behavior is attributed to the presence of the phenol and anhydride surface groups. In contrast, an interesting result is obtained in the CO emission of A-NaOH sample. In particular, two different peaks are shown in Figure 7 that indicate clearly the modification of the surface nature after the Na-OH treatment. The first peak is obtained between 600 and 800 °C and attributed to the weak acidic and neutral groups, such as ether and carbonyl. The second peak is obtained above 800 °C and is attributed to the basic groups, such as chromen, pyrone, and ketone [31]. 

Table 1 provides quantitative results obtained by integration of the TPD curves shown in Figure 6 and Figure 7. As shown in Table 1, the amount of CO desorbed seems quite similar for the different samples. However, the nature of the surface groups differs between the samples. Furthermore, the amount of CO_2_ decreases after NaOH treatment due to the strong acidic groups neutralisation. In contrast, the thermal treatment eliminated the main oxygen surface groups. 

The results obtained by Boehm titration are reported in Table 2. The values indicate that the sample A has only an acidic character. This is due to the use of phosphoric acid as an activating agent. Moreover, sample A is characterized by a low content of lactones, and a much higher amount of phenol and carboxylic groups. The treatment with nitric acid considerably enhanced the number of oxygenated acidic surface groups such as carboxyl, lactone, and phenol, as already observed by TPD-MS. Due to the presence of residual NaOH, no Boehm titration could be performed on the A-NaOH sample. In addition, no significant amounts were detected for the A-TT sample.

**Figure 8:**
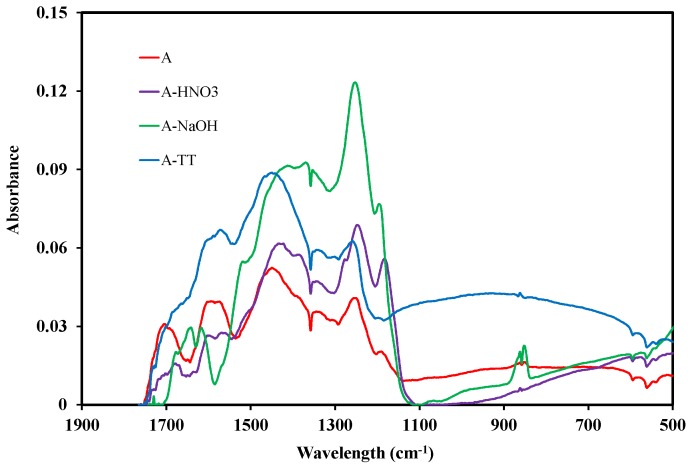
Fourier transformed infrared spectroscopy (FTIR) spectra of the different activated carbons prepared in the present study.

Fourier transformed infrared spectroscopy (FTIR) was used to characterize the main functional surface groups of the activated carbon. The 500–1900 cm^−1^ range of the FTIR spectra of the different samples are represented in Figure 8.

Various bands and peaks were observed for the different samples. A broad and intense shoulder between 3000 and 3500 cm^−1^ (not shown on Figure 8) was observed for samples A and A-HNO_3_, and was associated to the stretching vibrations of the hydroxyl groups of water adsorbed on the surface [35]. The band centered around 1700 cm^−1^ is ascribed to the stretching vibrations of C=O bond in the carboxylic acid and lactone groups [36]. This band was also detected for the A and A-HNO_3_ samples. The peaks observed between 1580 and 1700 cm^−1^ are attributed to the elongation vibration of the C-O group in the oxygenated surface groups such as lactone, carboxylic acid, and quinone [37,38]. These peaks were detected for all the samples. The peak around 1640 cm^−1^ for A-NaOH sample may be attributed to a C=O bond of quinone rather than carboxylic acid. The band at 1250 cm^−1^ is assigned to C-O stretching and O-H bending modes of alcoholic, phenolic and ether groups [39].

This band is also obtained for the different samples and increased sharply for A-NaOH samples.

The comparison between the different samples is complicated due to the variability in the results available in the literature concerning the peaks and their corresponding surface groups. In this present investigation, the FTIR spectra of A and A-HNO_3_ samples (Figure 8) shows similar shape. However, the intensity of the peaks are more pronounced for the A-HNO_3_ sample. Such a result indicates that the treatment of sample A with nitric acid (sample A-HNO_3_) increases the amount of surface chemical functionalities such as the carboxylic, lactones, and phenols groups without strongly modifying their nature. In contrast, the NaOH and heat treatments seem to modify the nature of the surface groups of the sample activated carbons. In particular, the peaks around 1700 cm^−1^, attributed to the C=O stretching in carboxylic and lactones groups, as well as the peak towards 3050 cm^−1^ attributed to the elongation of the O-H groups present in the carboxylic acids, disappeared after the NaOH and heat treatments. Therefore, NaOH and heat treatments play an important role in the removal of strong acidic groups. Furthermore, a significant increase of the phenol and ether groups was observed with the NaOH treatment through the sharp peak observed around 1250 cm^−1^. 

The present analysis of the surface chemistry evolution of the different activated carbons after the chemical and thermal treatments is very important to identify the interaction mechanism of NO_2_ with AC in the following sections.

### 3.2. NO_2_ Adsorption on the Activated Carbon A

**Figure 9:**
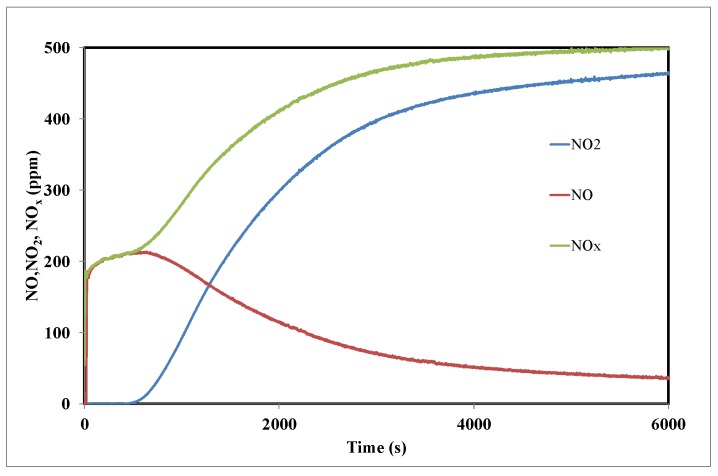
Outlet concentrations of NO_2_, NO and NO_x_ during the adsorption of 500 ppm NO_2_ at room temperature on the activated carbon A.

Figure 9 shows the out-streaming concentrations of NO and NO_2_ gases recorded during NO_2_ adsorption tests performed on 100 mg of the activated carbon (A) at ambient temperature with 500 ppm NO_2_ concentration feeding gas. The NO_x_ curve represents the cumulative concentrations of NO and NO_2_. 

As soon as the adsorption test started, a sharp increase of NO emission was observed. Then the NO concentration reached a plateau at concentration values higher than 200 ppm, and only after 16 min of stabilization could the emission of NO_2_ be recorded. During the first minutes of contact between the surface of the activated carbon and NO_2_, part of NO_2_ is reduced at the surface of the material into NO until stabilization at 200 ppm, the rest of NO_2_ seems then to be adsorbed at the surface until partial saturation of the surface. Then, after 600 s of experiment the slope of the breakthrough curve of NO_2_ increases. The total NO_2_ adsorption capacity measured for the activated carbon corresponded to 64.5 mg/g, and it was obtained when the surface was saturated by NO_x_, and after 6000 s. This adsorption capacity is lower than those obtained for physically activated carbons prepared from date pits [10] and olive stones [27], which correspond to 129 mg/g and 131 mg/g, respectively. Such a difference may be attributed to the presence of a high amount of acidic groups on the activated carbon surface. Belhachemi et al. [10] have observed a decrease in the adsorption capacity of commercially activated carbon after wet oxidation treatment.

**Figure 10:**
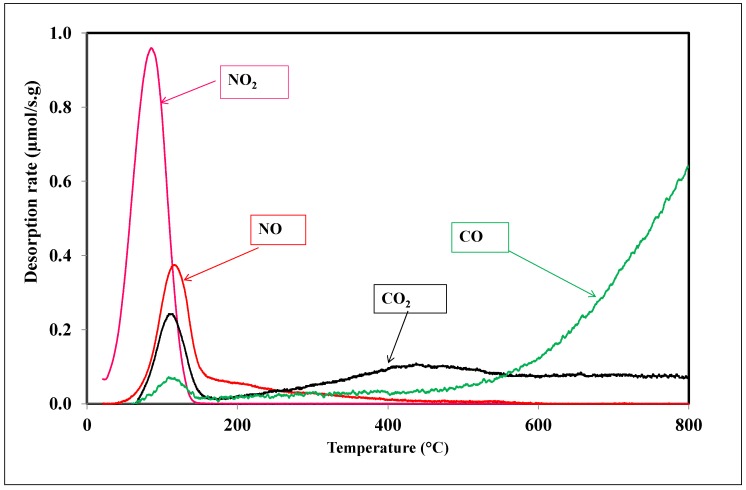
Emission of NO_2_, NO, CO, and CO_2_ observed during the TPD experiment carried out under nitrogen flow after saturation of the activated carbon A surface by NO_2_.

At the end of the adsorption step, a temperature-programmed desorption analysis was performed under nitrogen stream by heating the sample from room temperature to 800 °C at a heating rate of 5 °C/min. NO_2_, NO, CO_2_ and CO emissions were recorded as a function of temperature and shown in Figure 10. During the TPD experiment, several peaks were observed at different temperature intervals. Such behavior confirms the formation of various surface species during the adsorption of NO_2_.

Figure 10 shows that the desorption of NO_2_ stars at a low temperature, reaching a maximum at 78 °C and ending at 120 °C. This emission at low temperature is attributed to physisorbed NO_2_. The integration of this curve, as mentioned in Section 2.5, allows for the quantification of the amount of physisorbed NO_2_ during the adsorption tests (Table 3). 

The analysis of NO emission shows that it occurs at the same time than the CO and CO_2_ releasing. The NO emission started at 60 °C and reached a maximum at 120 °C, and the peak is superposed to those of CO and CO_2_. Therefore, the emissions of CO and CO_2_ are related to the chemisorption of NO_2_ on the surface of the activated carbon. Indeed, the formation of surface complexes were already mentioned by Jeguirim et al. [23], which decompose according to Equations (5) and (6):

(5) −C(ONO)→CO+ NO

(6) $$-C\left({\mathrm{ONO}}_{2}\right)\to {\mathrm{CO}}_{2}+\;\mathrm{NO}$$

The integration of the NO_2_ and NO curves permits the quantification of the amounts of physisorbed and chemisorbed NO_2_ (51.6 and 12.9 mg/g, respectively), and, to the best of our knowledge, this approach and quantification are for the first time here presented and they have never been previously applied in the literature. In fact, no other studies investigating the TPD step after NO_2_ adsorption on activated carbons were ever reported.

### 3.3. Effect of Surface Modification on the Adsorption Performances of Activated Carbons

#### 3.3.1. Effect of Acidic Group Incorporation

**Figure 11:**
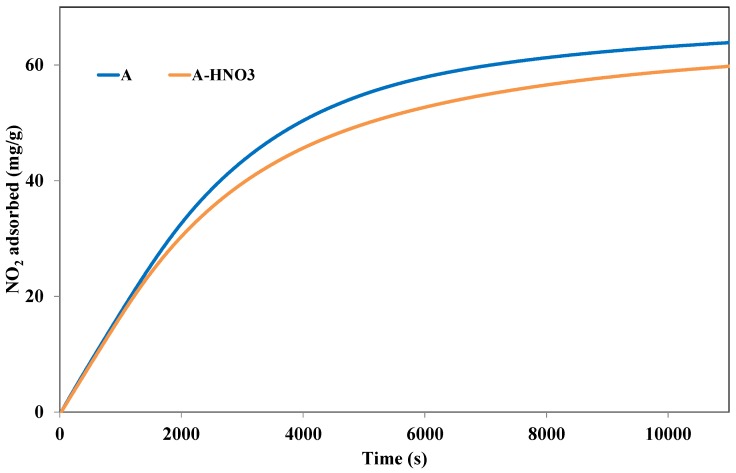
Evolution of the amount of NO_2_ adsorbed at the surface of the activated carbon treated with HNO_3_ and the activated carbon A, at 25 °C and until saturation.

In order to further understand the influence of the activated carbon acidic surface species on the adsorption of NO_2_, the activated carbon treated with nitric acid was tested in NO_2_ adsorption and compared to sample A. Indeed, the A-HNO_3_ sample presents a higher quantity of acid groups at the surface than sample A. Figure 11 shows the cumulative adsorption capacities of NO_2_ at 25 °C for the different activated carbons treated or not with HNO_3_. 

The comparison between the different samples shows a decrease in the adsorption capacities by treatment with HNO_3_. Such results clearly indicate that the lowest adsorption capacity is obtained for activated carbons possessing more carboxylic, lactone, and phenol surface functional groups. However, it is important to follow the evolution of the textural properties with the treatment of nitric acid. Analyses of these properties showed that oxidation by HNO_3_ has induced a decrease of the specific surface area of the activated carbon from 1175 m^2^/g for sample A to 971 m^2^/g for sample A-HNO_3_. Similarly, a decrease in the microporous volume was observed (from 0.45 cm^3^/g for sample A to 0.40 cm^3^/g for sample A-HNO_3_). In addition, the decrease of microporous volume may also explain up to a certain extent the decrease of the adsorption capacity after the HNO_3_ treatment. 

As expected, the treatment of the activated carbon surface with nitric acid shows that it did not promote the adsorption of NO_2_. In order to separate both effects (surface chemistry, micropores volume) on the adsorption capacity, we have compared the amounts of physisorbed and chemisorbed NO_2_. For these calculations, we have made the assumption that NO released during the TPD corresponds to the chemisorbed NO_2_ species, while the emitted NO_2_ corresponds to the physisorbed NO_2_. The amounts of physisorbed and chemisorbed NO_2_ were calculated by integration of the NO_2_ and NO curves, respectively, as previously explained. The obtained values are presented in Table 3.

The part of NO_2_ chemisorbed decreases slightly from 12.9 mg/g to (sample A) to 11.9 mg/g (sampleA-HNO_3_). Therefore, the addition of acidic groups on the activated carbon surface may inhibit the chemisorption process. Such behavior is in agreement with the previous investigations of Belhachemi et al. [10]. Authors found that the presence of carboxylic groups may inhibit the reduction of NO_2_ into NO, which is crucial step for NO_2_ adsorption. Table 3 also shows that the amount of physisorbed NO_2_ decreases significantly from 51.6 to 45.3 mg/g after the acidic treatment. This decrease seems to be correlated to the decrease of the micropores volume from 0.45 to 0.40 cm^3^/g. This information leads to the assumption that the physisorbed part of NO_2_ is located directly in the microporosity of the activated carbon. The physisorbed NO_2_ species may be blocked inside the micropores by steric effect, the thermal agitation may be sufficient to extract these molecules. According to Equations (5) and (6), the chemisorbed part of NO_2_ interacts with C* and C*O surface groups to form surface complexes (C* represents carbon atoms coming from the activated carbon structure). The treatment with HNO_3_ gives rise to the formation of new surface groups. However, the same quantity of C* and C*O surface groups seems to be available for chemisorption when comparing to parent activated carbon A. These stable groups are strongly anchored on the surface of the activated carbon, but their location and properties are not yet explained.

#### 3.3.2. Effect of NaOH Treatment

**Figure 12:**
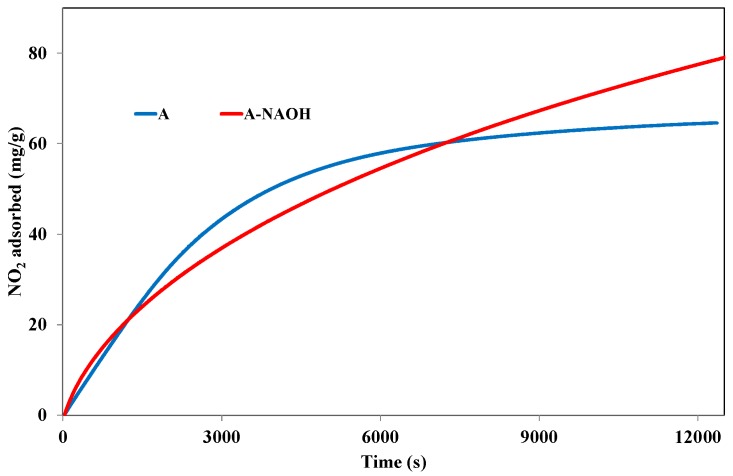
NO_2_ adsorption capacities at 25 °C for the activated carbons A and A-NAOH.

In order to further understand the influence of the activated carbon surface properties on the adsorption of NO_2_, the activated carbon A treated with NaOH was also tested in NO_2_ adsorption tests. Figure 12 shows the comparison between the evolutions of the adsorption capacities for samples A and A-NaOH. Clearly, the adsorption capacities increased from 64.5 mg/g for sample A to 79.1 for sample A-NaOH. This increase may be attributed to the formation of basic groups at the surface of the activated carbon after the NaOH treatment or to the partial neutralization of acid groups that, as previously showed, have a negative effect on the NO_2_ adsorption capacity. Moreover, the treatment with NaOH may generate free oxygen sites (C*O) available for NO_2_ chemisorption.

In order to further analyze these results, the chemisorbed and physisorbed amounts during both adsorption tests have been determined, and reported in Table 4. 

Table 4 shows that the amount of NO_2_ chemisorbed at the surface of the activated carbon after the alkaline treatment has significantly increased from 12.9 to 36.2 mg/g. This behavior confirms the role of NaOH on the availability of free oxygen surface groups as well as on the increasing amount of basic groups such as pyrone, ketone, quinone, and chromene. These surface oxygen groups are therefore available for the chemisorption of NO_2_. The chemisorption may be preceded by the reduction of NO_2_ into NO on the freely available free oxygen surface groups, as indicated by Jeguirim et al. [23]. Also, chemisorption could occur directly through the interaction with the basic surface groups. Such behavior is confirmed through a decrease in the reduction of NO_2_ into NO after the NaOH treatment. In fact, the ratio between the part of NO emitted and the part of NO_2_ adsorbed during the adsorption tests range from 1.05 (NO emitted: 1.48 mmol/g, NO_2_ adsorbed: 1.40 mmol/g) for sample A to 0.55 for sample A-NaOH (NO emitted: 0.96 mmol/g, NO_2_ adsorbed: 1.72 mmol/g). Such an inflection, attributed to the basic character of sample A-NaOH, was observed by Bashkova et al. [25]. Furthermore, Table 4 indicates that the physisorbed part of the NO_2_ amount also decreases significantly from 51.6 mg/g to 42.9 mg/g after NaOH treatment. This behavior can be attributed to the decrease of the micropores volume for A-NaOH as for the activated carbons treated with HNO_3_. 

#### 3.3.3. Effect of Acidic Surface Groups Elimination through Thermal Treatment

**Figure 13:**
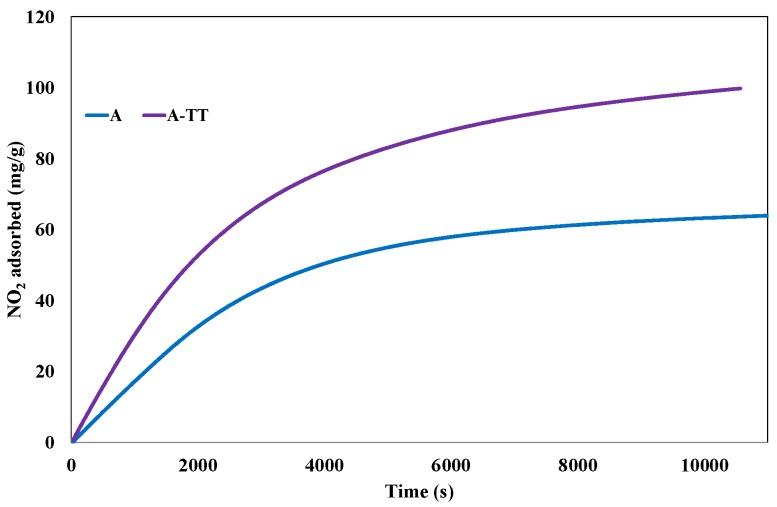
Evolution of the quantity of NO_2_ adsorbed at 25 °C on the surface of samples A and A-TT until saturation.

The adsorption capacity of the thermal treated activated carbons (A-TT) is compared with reference activated carbon A. The cumulative adsorption capacities of NO_2_ obtained with these two samples are presented in Figure 13. 

The thermal treatment leads to an increase of the amount of NO_2_ adsorbed at the saturation from 64.65 mg/g to 104 mg/g, although the micropores volume decreased only very slightly from 0.45 cm^3^/g to 0.44 cm^3^/g. Similar behavior was observed by Pietrzak et al. [11] during the thermal treatment of commercial activated carbon BAX-1500 at 950 °C for 60 min. The authors showed that this type of treatment increases the adsorption capacity from 42.7 to 50.5 mg/g. They have attributed this enhancement of the adsorption capacity to an increase of the basicity of the activated carbon.

Furthermore, as observed for A-NaOH, the ratio between the part of NO emitted and the part of NO_2_ adsorbed decreased from 1.05 for sample A to 0.85 for sample A-TT (NO emitted: 1.84 mmol/g, NO_2_ adsorbed: 2.26 mmol/g). Such behavior confirms that increasing the basicity of the activated carbon surface leads to the decrease of NO_2_ reduction to NO on the activated carbon surface.

### 3.4. Role of the Surface Oxygen Groups and the Textural Properties on the Interaction of NO_2_ with the Activated Carbon Surface

**Figure 14:**
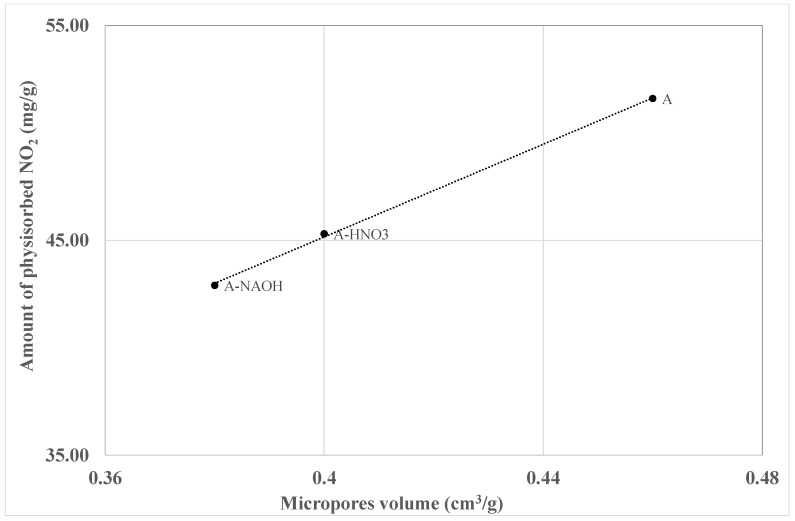
Correlation between the micropores volume and the amount of physisorbed NO_2_ on the different activated carbons.

The analysis of the different results that were presented in this study clearly indicates the role of the textural properties and the surface chemistry of the activated carbons on the adsorption mechanism of NO_2_. In particular, a good correlation can be established between the decrease of the physisorbed amount of NO_2_ and the microrpores volume of the different activated carbons (Figure 14).

In addition, it is observed that the elimination of surface oxygen groups leads to an increase of the chemisorbed NO_2_. These findings support the propositions of Radovic et al. [40], namely that the sites responsible for chemisorption are oxygen-free sites located at the edges of the graphene layers, whereas physisorption occurs all over the surface and in the pores. The increase of the surface basicity of the activated carbons reduce the NO emissions during the interaction of NO_2_ with activated carbons. Therefore, we may conclude that developed microporosity and the presence of an oxygen free site, as well as the basic surface groups, are the key parameters for the adsorption of NO_2_ on activated carbons.

## 4. Conclusions

Several investigations have examined NO_2_ adsorption on activated carbons prepared from lignocellulosic biomass precursors. However, the key factors that affect the interaction mechanism of NO_2_ with activated carbons are still not clearly identified. Therefore, in this study, activated carbons with different textural properties and surface chemistry were prepared. Then, the adsorption of 500 ppmv NO_2_ on the different activated carbons was examined in a fixed bed reactor at ambient temperature. Furthermore, temperature programmed desorption was applied after the adsorption tests in order to quantify the amount of the physisorbed and chemisorbed NO_2_.

Results showed different adsorption capacities for the different activated carbons, indicating the role of the surface chemistry and textural properties in the NO_2_ interaction mechanism. In particular, the presence of strong acidic groups on the activated carbon surface affect mainly the NO_2_ physisorption process. In fact, these surface oxygen groups that may block the NO_2_ access to the micropores.

In contrast, the NO_2_ chimisorption is strongly influenced by the presence of oxygen free sites and the presence of basic groups. The oxygen free sites allow the reduction of NO_2_ into NO, which leads to the formation of –C(O) groups. The NO_2_ may be chemisorbed on these –C(O) groups or on groups already having one oxygen, such as basic groups.

## Figures and Tables

**Table 1:** The amount of gases emitted during the temperature programmed desorption (TPD) experiments carried out with sample A, samples of A treated by HNO_3_ or NaOH and sample A thermally treated.

Carbons	CO_2_ (µmol/g)	CO (µmol/g)	H_2_O (µmol/g)	H_2_ (µmol/g)
A	719	3430	3460	1800
A-HNO	1560	3770	2160	1310
A-NaOH	217	3190	3300	4410
A-TT	0.25	3.04	1.98	2.05

**Table 2:** Quantification of the chemical surface groups (meq·g^−1^) by Boehm titration.

Sample	Carboxyl	Lactones	Phenols	Total Acid Sites	Total Basic Sites
A	1.45	0.05	0.7	2.20	0
A-HNO_3_	2.00	0.50	1.10	3.60	0.15

**Table 3:** Chemisorbed, physisorbed, and total amounts of NO_2_ adsorbed at the surface of the activated carbons treated or not with HNO_3_.

Sample	A	A-HNO_3_
NO_2_ adsorption capacities (mg/g)	64.5	57.2
NO_2_ physisorbed (mg/g)	51.6	45.3
NO_2_ Chemisorbed (mg/g)	12.9	11.9

**Table 4:** Chemisorbed, physisorbed and total amounts of NO_2_ adsorbed at the surface of the activated carbons treated or not with NaOH.

Sample	A	A-NaOH
NO_2_ adsorption capacities (mg/g)	64.5	79.1
NO_2_ physisorbed (mg/g)	51.6	42.9
NO_2_ chemisorbed (mg/g)	12.9	36.2
